# Study on Structural Performance of Asphalt Concrete and Hot Rolled Sheet Through Viscoelastic Characterization

**DOI:** 10.3390/ma13051133

**Published:** 2020-03-04

**Authors:** Senja Rum Harnaeni, Florentina Pungky Pramesti, Arif Budiarto, Ary Setyawan, Muhammad Imran Khan, Muslich Hartadi Sutanto

**Affiliations:** 1 Doctoral Program in Civil Engineering Department, Faculty of Engineering, Universitas Sebelas Maret, Surakarta 57162, Indonesia; 2 Civil Engineering Department, Universitas Muhammadiyah Surakarta, Surakarta 57169, Indonesia; 3 Civil Engineering Department, Universitas Sebelas Maret, Surakarta 57162, Indonesia; pungkypramesti@ft.uns.ac.id (F.P.P.); arifbudiarto@staff.uns.ac.id (A.B.); arysetyawan@staff.uns.ac.id (A.S.); 4 Civil & Environmental Engineering Department, Universiti Teknologi PETRONAS, Seri Iskandar 32610, Malaysia; Muhammad_17007177@utp.edu.my (M.I.K.); Muslich.sutanto@utp.edu.my (M.H.S.)

**Keywords:** dynamic modulus, phase angle, master curve, asphalt mixtures, asphalt concrete, hot rolled sheet

## Abstract

The aim of this study is to assess the viscoelastic parameters (i.e., phase angle and dynamic modulus) of asphalt concrete-wearing course (AC-WC) and hot rolled sheet-wearing course (HRS-WC) mixtures obtained from the dynamic modulus test. This study was accomplished in four stages: determining optimum asphalt content using Marshall mix design procedure, stability and flow parameters from Marshall test, viscoelastic parameters from dynamic modulus testing and finally the generation of dynamic modulus master curves at a reference temperature of 25 °C. The results showed that at the same temperature, the dynamic modulus of AC-WC and HRS-WC mixtures tended to increase with escalating the loading frequency, while dynamic modulus decreases with an increase in the test temperature at constant loading frequency. Furthermore, the dynamic modulus of the AC-WC mixture was recorded as 100% higher than the HRS-WC asphalt mixture. The phase angle, however, showed contradictory behavior with that shown in dynamic modulus. The phase angle of the AC-WC mixture and HRS-WC asphalt mixture showed almost the same behavior. Similarly, the dynamic modulus master curves of AC-WC and HRS-WC asphalt mixtures can be used to predict the dynamic modulus at the frequency range of 0.01 to 10 Hz and a reference temperature of 25 °C. The results were also used to evaluate the rutting and fatigue performance of AC-WC and HRS-WC.

## 1. Introduction

Wearing course from asphalt concrete and hot rolled sheet have different gradations, i.e., well-graded and gap-graded, respectively. Asphalt mixture is a viscoelastic material, which has viscous and elastic behavior depending on temperature and time/or loading frequency [1,2,3,4,5,6,7].

The viscoelastic characteristic of asphalt mixtures can be ascertained using dynamic modulus testing [6,8,9]. One of the dynamic modulus testing is the Asphalt Mixture Performance Tester (AMPT) in accordance with American Association of State Highway and Transportation Officials (AASHTO) 2015 [10], which uses a sinusoidal loading pattern with axial loading. For linear viscoelastic materials, the relationship between stress and strain is demonstrated by complex dynamic modulus (|E*|) when subjected to sinusoidal loading. The absolute value of the complex modulus is generally called the dynamic modulus [6,11,12,13,14,15,16].

The dynamic modulus master curves based on the reference temperature are used to calculate the effect of temperature and loading rate. With the availability of the master curve, dynamic modulus can be calculated at the desired loading frequency and temperature. Based on research results at the University of Maryland, dynamic master modulus curves that represent can be created using sigmoidal equations [16,17,18,19,20,21,22,23].

One of the equations used to create a master curve is the equation proposed by AASHTO. To estimate the reduced frequency the equations are known as the Arrhenius equation [24,25,26], and Williams–Landel–Ferry (WLF) equation [27] can be used. A tool that can be used to solve the master curve is the sum of the square error (SSE) method with optimization techniques and solver functions in Microsoft Excel software [28,29].

The purpose of this study is to assess the rutting and fatigue performance of AC-WC and HRS-WC using viscoelastic parameters (i.e., dynamic modulus and phase angle) obtained from Asphalt Mixture Performance Tester (AMPT) and to generate the dynamic modulus master curves that will be useful for design purposes.

## 2. Materials and Methods

**Figure 1:**
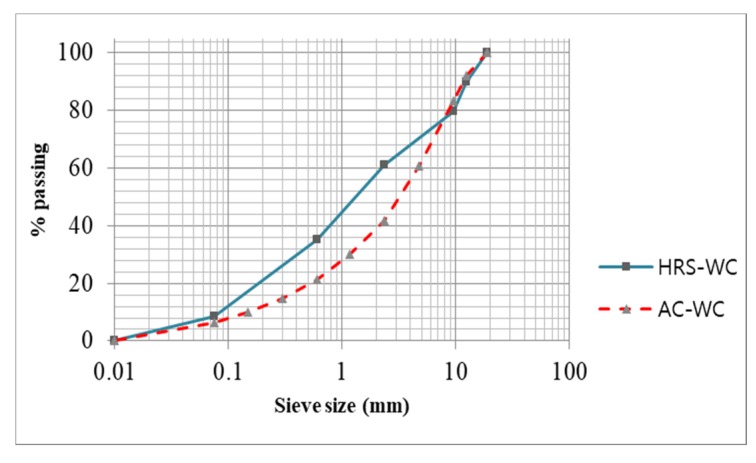
Aggregate gradations for AC-WC and HRS-WC mixtures.

The materials used in this study were locally available asphalt of penetration grade 60/70, coarse and fine aggregates that fulfilled the requirement of Bina Marga 2010 specifications [30]. The physical properties of asphalt, fine aggregate and coarse aggregate are given in Table 1, Table 2 and Table 3, while the aggregate gradation of asphalt concrete (AC) and hot rolled sheet (HRS) wearing courses are illustrated in Figure 1.

The test consisted of four stages. The first stage was to obtain optimum asphalt content from AC and HRS mixtures by the Marshall stability test. The second stage was to determine the Marshall properties of asphalt mixtures through the Marshall test; the third stage was to find viscoelastic parameters (i.e., phase angle and dynamic modulus) from dynamic modulus testing using AMPT and the four-stage was to generate master curve. Marshall test refers to AASHTO 2008 [31], and dynamic modulus testing using AMPT refers to AASHTO 2015 [10].

### 2.1. Specimen Preparation

**Figure 2:**
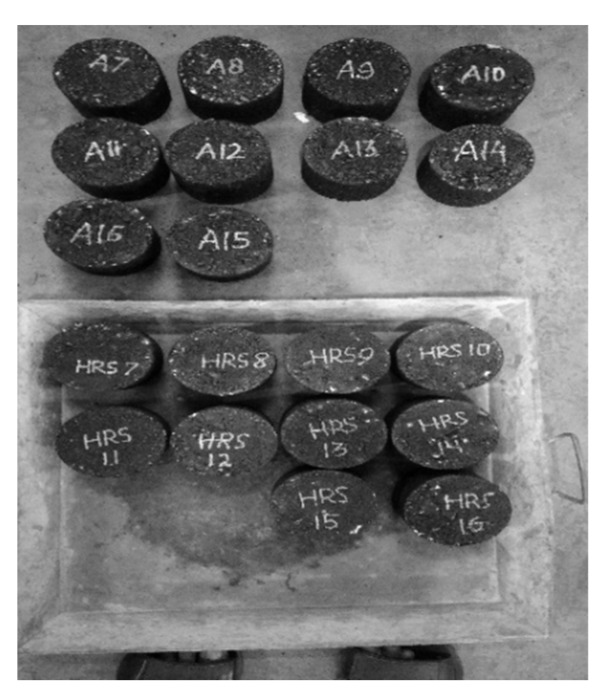
Specimens for the Marshall test.

**Figure 3:**
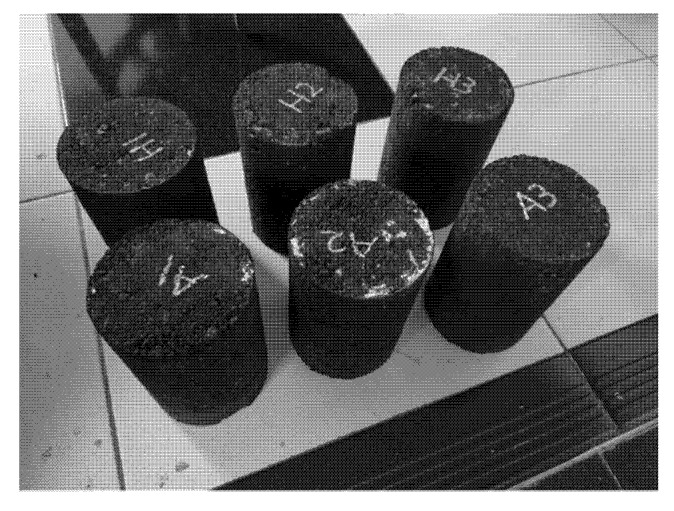
Specimens for dynamic modulus test.

Initially, Marshall samples with diameter of 100 mm and height of 63.5 mm were prepared with varying percentages of asphalt content for the determination of optimum asphalt content as per AASHTO specifications [31]. The asphalt content was selected as 4.5%–6.5% and 5%–7% by weight of total mix for AC and HRS asphalt mixtures, respectively. However, the specimens for dynamic modulus testing were prepared with height and diameter of 150 mm and 100 mm respectively in accordance with AASHTO specifications [10]. Each sample was prepared in triplicate from both type of mixtures. The specimens for the Marshall test and dynamic modulus test are presented in Figure 2 and Figure 3, respectively.

### 2.2. Marshall Stability Test

The Marshall test is used to determine the stability and flow of asphalt concrete mixtures. This test can be used to estimate the optimum asphalt content by determining stability and flow value in combination with the volumetric analysis. The maximum load that the specimen resists is presented by stability, whereas the deformation is measured as the flow of mixtures.

Initially, the Marshall stability test was performed on AC-WC mixtures with asphalt content of 4.5%, 5.0%, 5.5%, 6.0% and 6.5% to determine the corresponding stability and flow value. Similar tests were performed on HRS-WC specimens with asphalt content of 5%, 5.5%, 6.0%, 6.5% and 7.0%. The stability and flow values, in combination with corresponding volumetric properties, were used to obtain the optimum asphalt content of both mixtures. Furthermore, after determining the optimum asphalt contents, more mixtures were prepared using optimum asphalt content to determine the Marshall stability properties of both mixtures.

### 2.3. Dynamic Modulus Test

The dynamic modulus and phase angle of AC-WC and HRS-WC asphalt mixtures using Asphalt Mixture Performance Tester (AMPT) were determined by performing the dynamic modulus test. AMPT is a serve-hydraulic machine used to measure the engineering properties of asphalt mixtures. The dynamic modulus data were used to produce master curves for AC and HRS wearing course mixtures. The dynamic modulus test using AMPT used a sinusoidal loading pattern with axial loading. Range of temperatures (15, 25, 35 and 45 °C) and loading frequencies (10, 1, 0, 1, and 0.01 Hz) were used to carry out the dynamic modulus tests. The selected temperatures represent variations of temperature in Indonesia. Tests were conducted from the lowest temperature to high temperature and from high frequency to low frequency. The test was conducted on three specimens from each AC and HRS mixtures. The results of the dynamic modulus test were then used to develop master curves to predict the engineering properties of AC and HRS mixtures.

### 2.4. Generating Dynamic Modulus Master Curves

Based on data, dynamic modulus (|E*|) from the dynamic modulus tests, master curves were produced for AC and HRS asphalt mixtures at the reference temperature of 25 °C using the sum of square error (SSE) method by using the equation proposed by AASHTO. Furthermore, to solve the equation, the Arrhenius equation ([24], [25] and [26]), and Williams–Landel–Ferry (WLF) equation [27] can be used for calculation of shift factors. However, in this study, the Arrhenius equation was used to calculate the shift factor.

According to AASHTO 2015 [10], the dynamic modulus sigmoidal master curve equation in the Mechanistic-Empirical Pavement Design Guide (MEPDG) is:

(1) $$\log\left|E^{*}\right|=\delta +\frac{\left(\log{\left|E^{*}\right|}_{max}-\delta \right)\;}{1+\;e^{\beta +\gamma \log\;f_{r}}}$$

where |E*| is dynamic modulus (psi), *δ*, *β*, *γ* are sigmoidal equation constant, |E*| max is the maximum value of modulus (psi), *fr* reduced frequency (Hz) and log *fr* is Arrhenius equation as follows:

(2) $$\log f_{r}=\log f+\log\;\left[a\left(T\right)\right]=\log f+\;\frac{\Delta E_{a}}{19.14714}\left(\frac{1}{T}-\;\frac{1}{T_{r}}\right)$$

where *fr* is reduced frequency (Hz), *f* is loading frequency of at test temperature (Hz), $\Delta E_{a}$ is activation energy (constant), *T* is test temperature (°K), *Tr* is reference temperature (°K) and [a(T)] is shift factor at temperature *T* as the following:

(3) $$\log\;\left[a\left(T\right)\right]=\frac{\Delta E_{a}}{19.14714}\left(\frac{1}{T}-\;\frac{1}{T_{r}}\right)$$

The maximum modulus value $\;({\left|E^{*}\right|}_{\max)})$ is estimated from the volumetric properties of asphalt mixture using the Hirsch equation [16,32,33] and by limiting the maximum value of asphalt modulus of 1 GPa or 145,000 psi, as follows:

(4) $${\left|E^{*}\right|}_{max}=P_{c}\left[4,200,000\left(1-\frac{VMA}{100}\right)+435,000\;\left(\frac{VFA\;x\;VMA}{10,000}\right)+\frac{1-\;P_{c}}{\frac{\left(1-\;\frac{VMA}{100}\right)}{4,200,000}+\frac{VMA}{435,000\;\left(VFA\right)}}\right]$$

One tool that can be used to solve the master curve is the SSE method with optimization techniques and solver functions in MS Excel software. The SSE method is a spreadsheet optimization technique based on minimizing the SSE between E(f) measured and E(f) predicted estimated using the fit function [28]. In this method, the solver function in MS Excel is iteratively used to calculate the best values of *δ*, *β*, *γ* and $\Delta E_{a}$ in Equation (1) to create the master curves that best matches E(f) measured. The basic concept of this method is to obtain the most appropriate function for E(f) iteratively by changing *δ*, *β*, *γ*, and $\Delta E_{a}$ using the solver function in MS Excel so that the SSE between E(f) measured and E(f) predicted must close to zero. The log format for E(f) and frequency values can be used in an effort to shorten the equations. The SSE model formula is as follows:

Log l E(f)predicted l = Log l E(f) measured 1(5)

SSE = Σ (Log l E(f)predicted l − Log l E(f) measured l)2 ≈ 0.00(6)

## 3. Results and Discussion

### 3.1. Marshall Test

**Figure 4:**
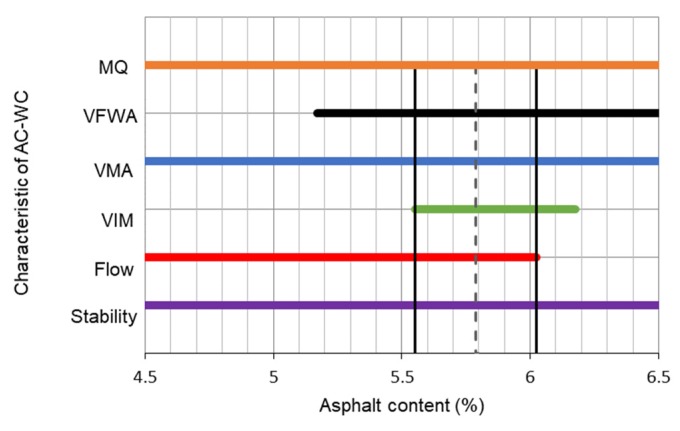
Optimum asphalt content of AC-WC mixture.

**Figure 5:**
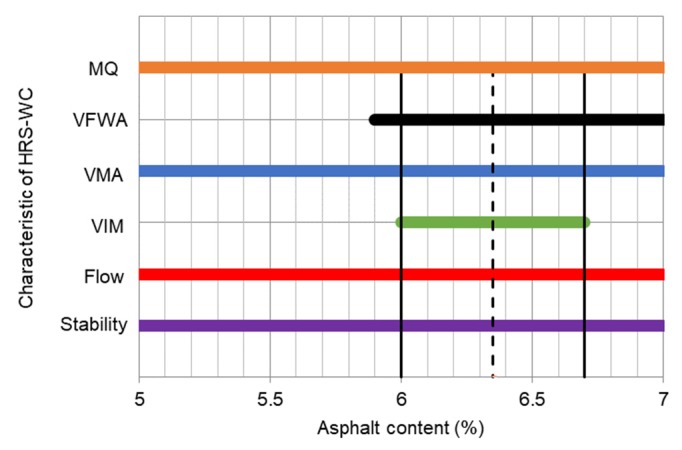
Optimum asphalt content of HRS-WC mixture.

Initially, the Marshall stability tests and volumetric analysis were conducted for determination of the optimum asphalt content for AC and HRS mixtures. The results are illustrated in Figure 4 and Figure 5. The optimum asphalt content of 5.8% and 6.35% were concluded for AC and HRS mixtures respectively. Furthermore, using the optimum asphalt content, the Marshall and volumetric properties of AC and HRS mixtures are demonstrated in Table 4.

### 3.2. Dynamic Modulus of AC-WC and HRS-WC Asphalt Mixtures

**Figure 6:**
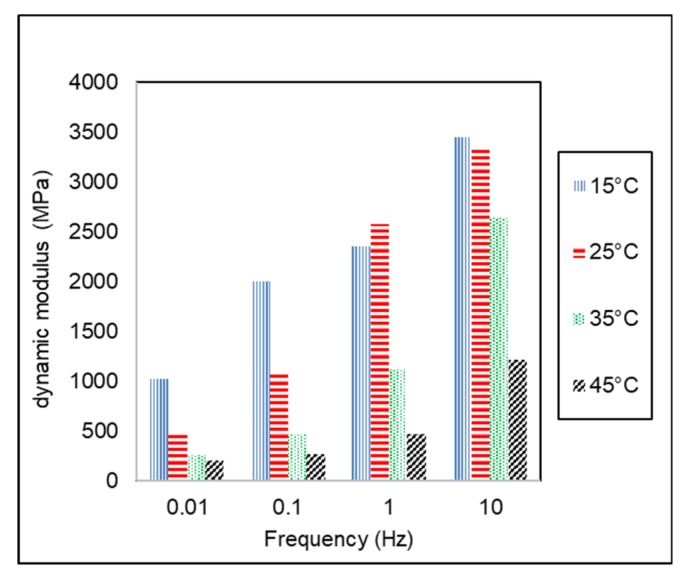
Variation of Dynamic Modulus of AC-WC mixture with temperature and frequency.

**Figure 7:**
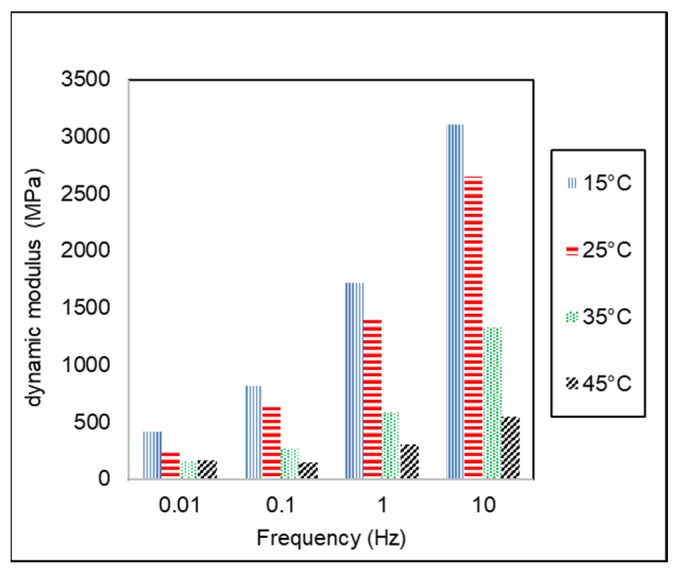
Variation of Dynamic Modulus of HRS-WC mixture with temperature and frequency.

Dynamic modulus (|E*|) of AC-WC and HRS-WC asphalt mixtures from dynamic modulus testing using AMPT are shown in Figure 6 and Figure 7. Asphalt is a viscoelastic material, and hence the behavior depends on temperature and time/or loading frequency. At low temperature and short time (or high speed), loading asphalt behaves as elastic, whereas at high temperatures and long-time (or slow-moving loads) the asphalt behaves as viscous material.

The viscoelastic characteristics of asphalt mixture can be determined using the dynamic modulus test and static creep test. The current study is limited to use the dynamic modulus test for the characterization of asphalt mixture properties. One of the dynamic modulus testing tools is the Asphalt Mixture Performance Tester (AMPT), referring to AASHTO 2015 [10], using sinusoidal loading patterns with axial loading. For linear viscoelastic materials, the relationship between stress and strain is demonstrated by complex dynamic modulus (|E*|) when subjected to sinusoidal loading.

Figure 6 and Figure 7 show the variation of dynamic modulus (|E*|) with temperature and frequency for AC and HRS asphalt mixtures. It can be seen from the results that increase in loading frequency causes an increase in the dynamic modulus at a reference temperature. It indicates that the rate of loading significantly influenced the asphalt mixture properties. Due to the higher dynamic complex modulus, the asphalt mixture will behave as elastic material at high loading frequency (faster loading rate). On the other hand, at a lower frequency (slow loading rate), the dynamic complex modulus is lower, and the mixture will behave as a viscous medium. Likewise, dynamic modulus tended to decrease with inclining test temperature at the same testing frequency. This trend is similar to another study conducted by Bayane et al. (2017) [34]. It shows that the temperature dependency influenced asphalt mixture as a viscoelastic material. Asphalt mixture is stronger at low temperatures due to higher dynamic complex modulus, as shown in Figure 5 and Figure 6. The temperature dependency of asphalt material is caused by the dominant viscous behavior at high temperatures, as well as dominant elastic behavior at low temperatures. Table 5 shows the comparison of dynamic modulus of AC and HRS asphalt mixtures at different combinations of temperature and frequencies. The results indicate that AC-WC asphalt mixtures have higher dynamic modulus than that of HRS-WC asphalt mixture at both conditions, i.e., at a low temperature-high frequency and high temperature-low frequency. The dynamic modulus of AC-WC mixture is around 100% higher than the dynamic modulus of HRS-WC asphalt mixture.

### 3.3. Phase Angle of AC-WC and HRS-WC Asphalt Mixtures

**Figure 8:**
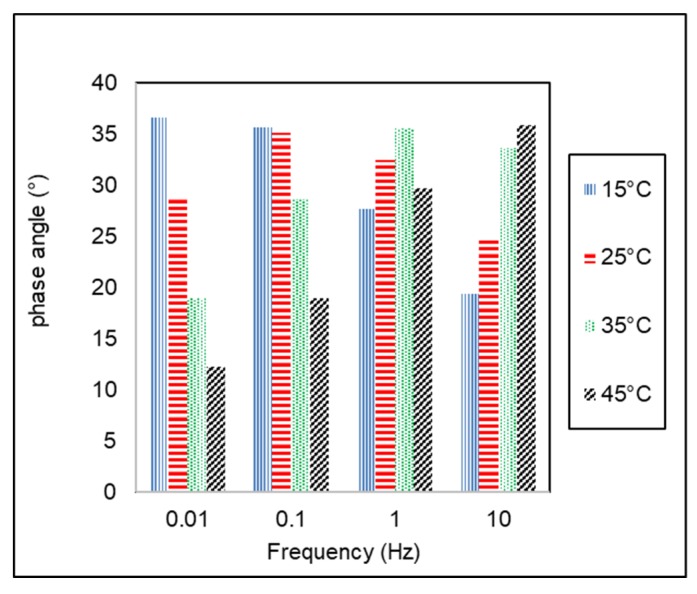
Variation of phase angle of AC-WC with temperature and frequency.

Phase angle (δ) of AC-WC and HRS-WC asphalt mixtures from dynamic modulus testing using AMPT are shown in Figure 7 and Figure 8. The angle between peak strain and stress is known as phase angle, indicating the viscoelastic characteristics of the asphalt mixtures. Ideal elastic and viscous material has a phase angle (δ) equal to 0° and 90° respectively, whereas the viscoelastic material has phase angle in between 0° to 90° (δ = 0° < δ < 90°).

**Figure 9:**
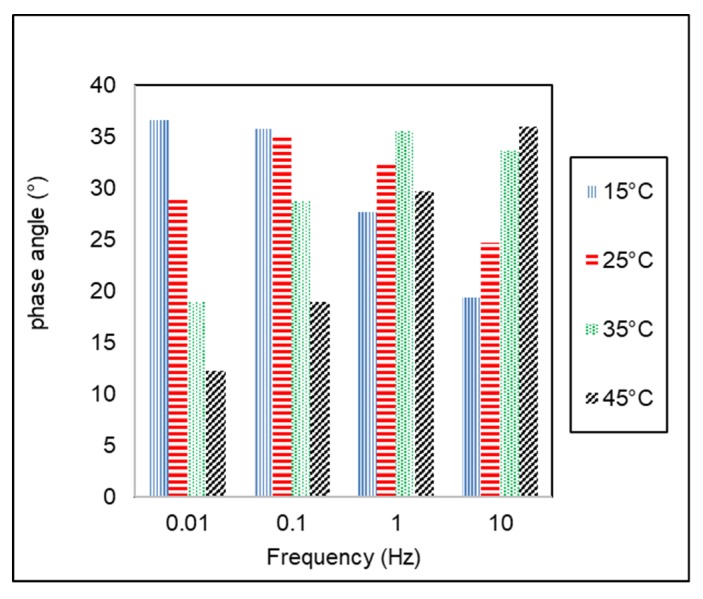
Effect of temperature and frequency on the phase angle of HRS-WC mixture.

Figure 8 and Figure 9 show the variation of phase angle (δ) with temperature and frequency for both type of asphalt mixtures. The phase angle tends to decrease with increasing loading frequency at the same temperature. On the other hand, it tends to increase with increasing temperature at the same testing frequency. This trend is similar to previous study conducted by Bayane et al., (2017) [34]. The AC-WC asphalt mixture shows lower value of phase angle as compared to HRS-WC asphalt mixture, which indicates that AC-WC mixtures are more elastic than HRS-WC asphalt mixtures.

According to Bhasin et al. (2004) [35] and Bayane et al. (2017) [34], dynamic modulus value ǀE*ǀ can be used to characterize the rutting and fatigue factors from expressions shown in equations 7 and 8 respectively.

Rutting factor = ǀE*ǀ/sin δ (7)

Fatigue factor = ǀE*ǀ × sin δ (8)

The higher complex modulus (ǀE*ǀ) and lower phase angle (δ) will have a higher value of rutting factor, which shows that the asphalt mixture has a high resistance to rutting, while the lower the value of fatigue factor indicates better resistance to fatigue. In this research, the comparison of the rutting factor of AC and HRS mixtures was investigated at a reference temperature of 25 °C and frequency of 1 Hz and 10 Hz, whereas the comparison of fatigue factor was investigated at a reference temperature of 15 °C and frequency of 1 Hz and 10 Hz because fatigue usually occurs in pavement with intermediate temperature. Frequencies of 1 Hz and 10 Hz were chosen to represent the lower and higher speed of vehicles, respectively [34,36].

From Table 6, the AC-WC mixtures have higher rutting factor than that of HRS-WC asphalt mixture at frequencies of 1 Hz and 10 Hz, which implies that the AC-WC mixture has better resistance to rutting as compared to HRS-WC asphalt mixture. It is because the dynamic modulus of the AC-WC mixture is much higher than that of HRS-WC asphalt mixture. In contrast, the fatigue factor of the HRS-WC asphalt mixture is smaller as compared to AC-WC mixture at frequencies of 1 Hz and 10 Hz. Hence, it can be concluded that HRS-WC asphalt mixture has better resistance to fatigue than that of AC-WC mixture.

### 3.4. Dynamic Modulus Master Curve of AC-WC and HRS-WC Asphalt Mixtures

The dynamic modulus master curve based on reference temperature is used to calculate the effect of temperature and loading rate. With the availability of the master curve, dynamic modulus value can be calculated at the referred loading frequency and temperature. Based on research results at the University of Maryland, dynamic master modulus curves that represent can be created using sigmoidal equations [16,17].

Master curves at the reference temperature were generated to investigate the effect of temperature and loading rate on the viscoelastic properties of the asphalt mixture. The master curve was constructed from dynamic modulus (|E*|), which was determined from the dynamic modulus test and carried out at a different temperature and loading frequencies. The viscoelastic material dependency temperature is defined by the number of shifts needed at each temperature to create a master curve. The dynamic modulus (|E*|) value of asphalt mixture produced from dynamic modulus test to generate the master curves, making shift factors according to equation (3) and to estimate the proposed master curve equation according to AASHTO equation (1) using the Arrhenius equation (2) to calculate reduced frequency.

**Figure 10:**
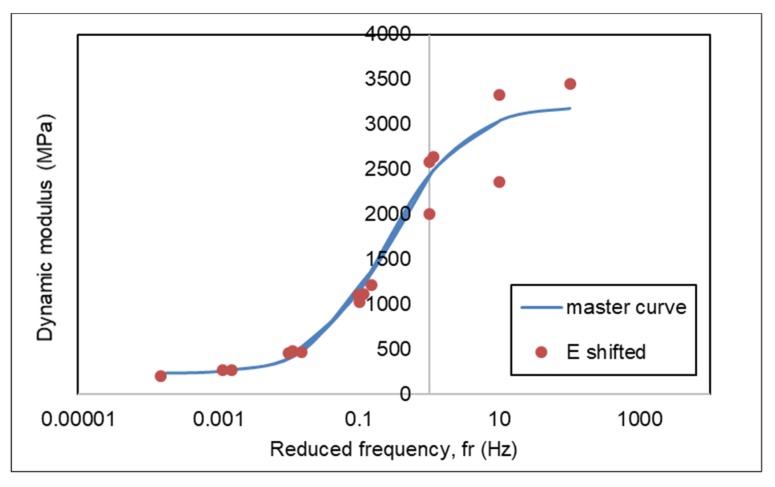
Master curve of AC-WC mixture.

**Figure 11:**
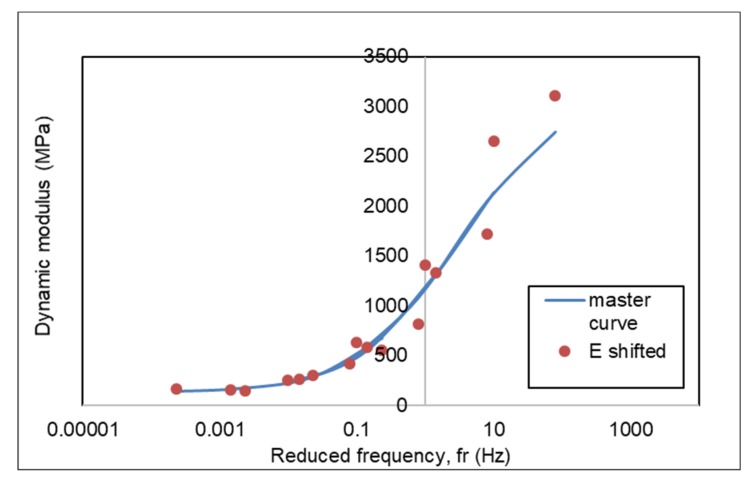
Master curve of HRS-WC mixture.

Based on the results of the dynamic modulus test, master curves were generated for AC and HRS mixtures at a reference temperature of 25 °C using the sum of square error (SSE) method. Table 7 and Table 8 show the dynamic modulus at a reference temperature of 25 °C for AC and HRS mixtures. The master curves generated for AC and HRS mixture are demonstrated in Figure 10 and Figure 11, respectively.

Constants δ, β, γ and $\Delta E_{a}$ in equation (3) for AC and HRS mixtures with the SSE method were obtained from Microsoft Excel using the solver function. By providing an initial value, the solver found the values of the constants δ, β, γ and $\Delta E_{a}$ to produce the E(f) _predicted_ results that are closest to the E (f) _measured_ value by minimizing the sum of square error values (SSE).

**Figure 12:**
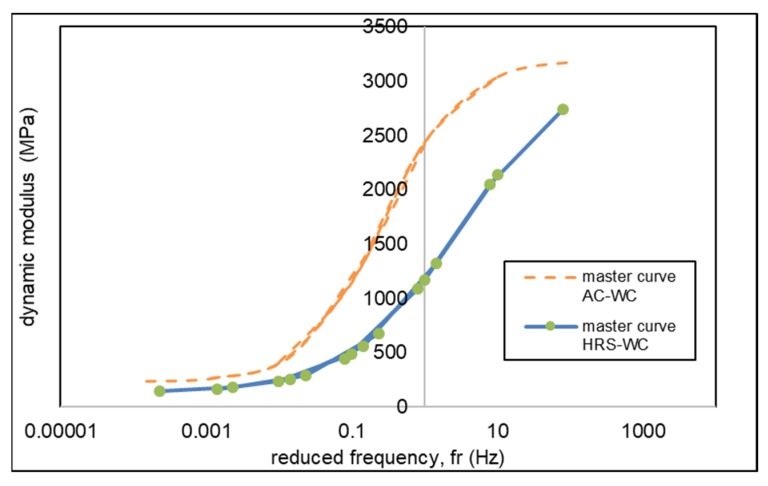
Comparison of master curves for AC-WC and HRS-WC mixtures.

Figure 12 presents the combine master curves for AC and HRS mixtures at a reference temperature of 25 °C. The dynamic modulus of AC-WC mixtures is higher than that of HRS-WC asphalt mixtures at a corresponding frequency and hence presenting higher resistance to rutting.

The results obtained in this study have the same trend as Suaryana’s study [37] and Nobakht’s study [20]. The AC mixtures demonstrate higher dynamic modulus as compared to HRS mixtures. The dynamic modulus of AC mixture is higher both at low and high temperature as compared with HRS mixture and hence, it shows that the AC-WC mixture provides more resistance when compared to the HRS-WC asphalt mixture.

## 4. Conclusions

The effect of different temperature and loading frequencies on AC-WC and HRS-WC mixtures were investigated using dynamic modulus and phase angle. Similarly, rutting and fatigue factors, as well as master curves, were generated to evaluate the effect of these mixtures on fatigue and rutting performance at a reference temperature. Following conclusions are observed from this study.

The dynamic modulus of AC-WC and HRS-WC mixtures tend to increase with increasing loading frequency at the same reference temperature, while dynamic modulus decreases with increasing test temperature at the same frequency. AC-WC mixtures have 100% higher dynamic modulus as compared to HRS-WC mixture, while the phase angle has a contradictory behavior with dynamic modulus. At the same temperature, the phase angle decreases with increasing loading frequency, while at the same loading frequency, the phase angle tends to increase along with increasing test temperature for both AC-WC and HRS-WC mixtures.

The AC-WC mixture presents higher resistance to rutting due to higher dynamic modulus and lower phase angle as compared to HRS-WC mixtures. However, HRS-WC mixture has better fatigue life in comparison with AC-WC mixture. The generated master curves also indicate that AC-WC mixture has a better higher dynamic modulus at reference temperature and frequency when compared with HRS-WC mixtures.

## Figures and Tables

**Table 1:** Physical properties of asphalt penetration 60/70.

Physical Property	Specification	Value
Penetration at 25 °C (0.1 mm)	60–79	66
Softening point (°C)	≥48	53.5
Ductility (mm)	≥100	≥100
Flash point (°C)	≥232	270
Specific gravity	≥1.0	1.07

**Table 2:** Physical properties of fine aggregate.

Physical Property	Specification	Value
Bulk Specific Gravity	-	2.59
SSD Specific Gravity	-	2.70
Apparent Specific Gravity	-	2.91
Water Absorption	≤5%	4.17%
Sand Equivalent	≥60%	96.6%

**Table 3:** Physical properties of coarse aggregate.

Physical Property	Specification	Value
Abrasion	≤30%	20.90%
Bulk Specific Gravity	-	2.49
SSD Specific Gravity	-	2.50
Apparent Specific Gravity	-	2.52
Water Absorption	≤3%	0.51%

**Table 4:** Marshall properties of AC-WC and HRS-WC asphalt mixtures.

Marshall Properties	Unit	AC-WC		HRS-WC	
		Spec	Actual	Spec	Actual
Marshall Stability	Kg	≥800	1412.31	≥800	1319.51
Flow	mm	2 ≤ 4	3.57	≥3	3.77
VIM (Void in the mix)	%	3 ≤ 5	4.54	4 ≤ 6	5.46
VMA (Void in mineral aggregate)	%	≥14	17.53	≥18	19.61
VFWA (Void filled with asphalt)	%	≥65	74.14	≥68	72.25
Marshall Quotient (MQ)	Kg/mm	≥250	402.03	≥250	356.37
Density	gr/cm^3^		2.32		2.30

**Table 5:** Dynamic modulus of AC-WC and HRS-WC mixtures at different temperature and frequency.

|E*|, MPa (Temperature 15 °C, Frequency 10 Hz)		|E*|, MPa (Temperature 45 °C, Frequency of 0.01 Hz)	
AC-WC	HRS-WC	AC-WC	HRS-WC
6780	3107	205	162

**Table 6:** Rutting and fatigue factors of AC-WC and HRS-WC mixtures at a reference temperature of 25 °C.

Rutting Factor = ǀE*ǀ/sin δ				Fatigue Factor = ǀE*ǀ × sin δ			
AC-WC		HRS-WC		AC-WC		HRS-WC	
f (Hz)		f (Hz)		f (Hz)		f (Hz)	
1	10	1	10	1	10	1	10
4795	11947	2623	6040	1384	2086	757	1161

**Table 7:** Dynamic modulus of AC-WC asphalt mixture at a reference temperature 25 °C (with ${\left|E^{*}\right|}_{max}$ = 465,592.1 kPa and constants: δ = 5.35, β = −2.1558, γ = −1.701 and ∆*E*a =165,238.8).

Test Temperature (°C)	f (Hz)	$\left|E^{*}\right|\;(\mathrm{MPa})$
15	10 1 0.1 0.01	3071.6 2939.3 2365.7 1122.9
25	10 1 0.1 0.01	2938.2 2361.3 1117.6 402.3
35	10 1 0.1 0.01	2419.2 1191.1 422.3 258.3
45	10 1 0.1 0.01	1343.3 468.2 266.3 231.2

**Table 8:** Dynamic modulus of the HRS-WC asphalt mixture at a reference temperature of 25 °C (with ${\left|E^{*}\right|}_{\max}$ = 464,566.9 kPa and constants: δ = 5.11, β = −0.7839, γ = −1.143 and $\Delta E_{a}$ = 148,300.6).

Test Temperature (°C)	f (Hz)	$\left|E^{*}\right|\;\left(MPa\right)$
15	10 1 0.1 0.01	2740.9 2047.9 1083.7 444.2
25	10 1 0.1 0.01	2135.4 1173.2 484.3 232
35	10 1 0.1 0.01	1322.5 557.9 254.0 166.0
45	10 1 0.1 0.01	676.3 291.3 176.8 143.8
